# Microglial HIV-1 Expression: Role in HIV-1 Associated Neurocognitive Disorders

**DOI:** 10.3390/v13050924

**Published:** 2021-05-17

**Authors:** Hailong Li, Kristen A. McLaurin, Jessica M. Illenberger, Charles F. Mactutus, Rosemarie M. Booze

**Affiliations:** Department of Psychology, University of South Carolina, Columbia, SC 29208, USA; hailong@mailbox.sc.edu (H.L.); mclaurik@email.sc.edu (K.A.M.); illenber@email.sc.edu (J.M.I.); mactutus@mailbox.sc.edu (C.F.M.)

**Keywords:** EcoHIV, HIV, HAND, microglia, RNAscope, viral reservoir

## Abstract

The persistence of HIV-1 viral reservoirs in the brain, despite treatment with combination antiretroviral therapy (cART), remains a critical roadblock for the development of a novel cure strategy for HIV-1. To enhance our understanding of viral reservoirs, two complementary studies were conducted to (1) evaluate the HIV-1 mRNA distribution pattern and major cell type expressing HIV-1 mRNA in the HIV-1 transgenic (Tg) rat, and (2) validate our findings by developing and critically testing a novel biological system to model active HIV-1 infection in the rat. First, a restricted, region-specific HIV-1 mRNA distribution pattern was observed in the HIV-1 Tg rat. Microglia were the predominant cell type expressing HIV-1 mRNA in the HIV-1 Tg rat. Second, we developed and critically tested a novel biological system to model key aspects of HIV-1 by infusing F344/N control rats with chimeric HIV (EcoHIV). In vitro, primary cultured microglia were treated with EcoHIV revealing prominent expression within 24 h of infection. In vivo, EcoHIV expression was observed seven days after stereotaxic injections. Following EcoHIV infection, microglia were the major cell type expressing HIV-1 mRNA, results that are consistent with observations in the HIV-1 Tg rat. Within eight weeks of infection, EcoHIV rats exhibited neurocognitive impairments and synaptic dysfunction, which may result from activation of the NogoA-NgR3/PirB-RhoA signaling pathway and/or neuroinflammation. Collectively, these studies enhance our understanding of HIV-1 viral reservoirs in the brain and offer a novel biological system to model HIV-associated neurocognitive disorders and associated comorbidities (i.e., drug abuse) in rats.

## 1. Introduction

Human immunodeficiency virus type 1 (HIV-1), which afflicts 38.0 million individuals globally [1], continues to be a public health crisis. Within three to five days of HIV-1 infection [2,3], infected monocytes and CD4+ cells transmigrate across the blood–brain barrier (BBB) [4] infecting astrocytes, perivascular macrophages, and microglial cells [5]. Although combination antiretroviral therapy (cART) suppresses viral replication in the periphery, it fails to penetrate the BBB [6]. Consequently, the central nervous system (CNS) acts as a viral reservoir for HIV-1 [7], a factor that is associated with the emergence of virus resistance [8,9] as well as the development and progression of HIV-1 associated neurocognitive disorders (HAND) [10,11].

Microglia cells are innate immune cells in the CNS that represent 5–20% of adult brain cells [12]. Key structural characteristics of microglia include a long half-life [13], ability to undergo cell division [13,14], and susceptibility to HIV-1 infection [15] support microglia as one of the viral reservoirs for HIV-1 in the brain [16]. Specifically, utilization of a highly sensitive in situ hybridization technique, in combination with immunohistochemistry, revealed that brain macrophages and microglia, but not astrocytes, were harboring HIV-1 DNA in the brain of HIV-1 aviremic patients [17]; results that are consistent with earlier findings in HIV-1 seropositive individuals with HIV-1 encephalitis [5,18]. Furthermore, aberrant microglial activation is associated with alterations in synaptic function [19], neurotransmitter excitotoxicity [20], and neuroinflammation [21]; functional alterations that have also been observed in HIV-1 seropositive individuals [22,23,24]. Altogether, microglia exhibit structural and functional characteristics that meet the criteria of a viral reservoir for HIV-1 infection in the brain [25].

Studying CNS infection of HIV-1 in humans, however, is limited by ethical and technical constraints, supporting the importance of biological systems to model key aspects of HIV-1 and HAND. Specifically, biological systems including macaques infected with Simian immunodeficiency virus [26,27,28], humanized mice [29], and EcoHIV infected mice [30] have provided strong evidence for the infection of microglial cells and development of a viral reservoir in HIV-1. However, to date, no study has systematically evaluated the major cell type expressing HIV-1 mRNA in the HIV-1 transgenic (Tg) rat, which has been supported as a biological system to model HAND in the post-cART era [31,32,33,34]. In light of these gaps in our knowledge, two complementary experiments were conducted to (1) evaluate the HIV-1 mRNA neuroanatomical distribution pattern and major cell type expressing HIV-1 mRNA in the HIV-1 Tg rat, and (2) validate our findings by developing and critically testing a novel model of HIV-1 infection in the rat (i.e., chimeric HIV (EcoHIV)).

First, the HIV-1 Tg rat, a biological system to model constitutive HIV-1 viral protein exposure, was utilized to characterize the brain regions and major cell type expressing HIV-1 mRNA. The HIV-1 Tg rat, originally reported by Reid et al. [35], resembles HIV-1 seropositive individuals on cART and has been utilized to investigate neurocognitive [31,32,34] and neuroanatomical [33,36,37] alterations associated with HAND. The functional deletion of *gag-* and *pol-,* a reverse transcriptase, precludes viral replication rendering the HIV-1 Tg rat noninfectious. HIV-1 viral transcripts have been observed throughout the body of the HIV-1 Tg rat including in the spleen, liver, thymus, lymph nodes, and kidneys [35,38,39]. Furthermore, viral proteins have also been detected in the tissues, macrophages, and serum of these animals [38,40]. Although the HIV-1 provirus is present in all cells, the actual HIV-1 mRNA expression pattern in the brain of the HIV-1 Tg rat is still unknown; a need that was addressed in the present study by utilizing RNA in situ hybridization (RNAscope), a highly sensitive and innovative technique.

Second, we developed a novel biological system to model systemic HIV-1 infection in the rat using EcoHIV. EcoHIV infection in mice, originally reported by Potash et al. [41], recapitulates many of the clinical features of HIV-1 commonly observed in humans including lymphocyte and macrophage infection, induction of antiviral immune responses, neuro-invasiveness, and an increase in inflammatory and antiviral factors in the brain [30,42]. Critically, the EcoHIV construct replaces the coding region of gp120 with gp80 from ecotropic murine leukemia viruses (MLV; [41]). The ecotropic MLV infects the host’s cells by binding to the cationic amino acid transporter (CAT-1) [43,44]. Previous studies have described the distribution of CAT-1 expression in the rat brain, whereby expression was observed throughout the brain including both the cortex and hippocampus [45]. Despite the significant utility of EcoHIV mice, extending this biological system to rats, which are more commonly utilized for studies of HAND and drug abuse, would be advantageous. Thus, the present study utilized in vitro and in vivo techniques to critically test and characterize EcoHIV infection in rats. Understanding HIV-1 viral reservoirs in the brain and the development of a novel biological system to assess HAND and associated comorbidities (i.e., drug abuse) may afford innovative targets for the improvement of therapeutic strategies.

## 2. Materials and Methods

### 2.1. Animals

All rats in the current experiments were pair-housed in AAALAC-approved facilities using the guidelines of the National Institutes of Health. The conditions were targeted at a 12 h:12 h light/dark cycle with lights on at 700 h (EST), 21 °C ± 2 °C, and 50% ± 10% relative humidity. Animals had ad libitum access to rodent chow (20/20X (Harlan Teklad, Madison, WI, USA)) and water. Fischer (F344/N) and HIV-1 Tg rats were obtained from Envigo Laboratories (Indianapolis, IN, USA). The project protocol was approved by the Institutional Animal Care and Use Committee (IACUC) at the University of South Carolina (federal assurance number: #D16-00028).

### 2.2. Experimental Design

**Figure 1:**
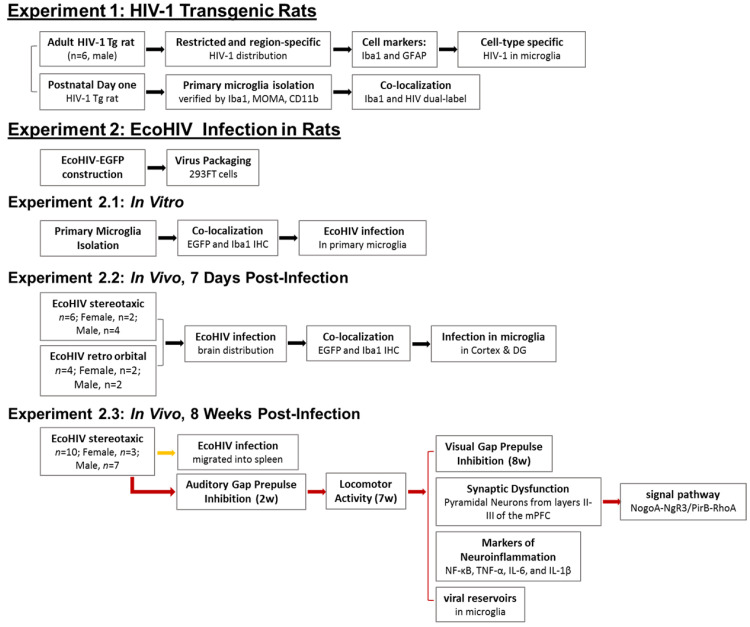
Schematic of the experimental design.

A schematic of the experimental design is presented in Figure 1.

### 2.3. Experiment 1

RNAscope In Situ Hybridization

RNAscope in situ hybridization was used to evaluate the expression of HIV-1 mRNA across different brain regions of HIV-1 Tg animals. The RNAscope in situ hybridization protocol was explained in detail by Li et al. [46].

In brief, HIV-1 Tg rats (*n* = 6) were deeply anesthetized using 5% sevoflurane and sacrificed. The rat brain was removed and frozen in liquid nitrogen within 5 min of sacrifice. Sagittal sections were sliced at 30 µm by cryostat and mounted onto SuperFrost Plus slides, which were dried at −20 °C for 10 min. Subsequently, slides were immersed in 4% paraformaldehyde at 4 °C for 1 h, followed by an increasing ethanol gradient (50%, 70%, 100% EtOH). Next, a pretreatment reagent (i.e., Protease IV Reagent, RNAscope Fluorescence Multiplex Kit, Advanced Cell Diagnostics, Newark, CA, USA) was applied to sections. Sections were incubated for 30 min and then hybridized with specific probes for HIV-1 viral proteins (see Supplementary Table S2). Slides were placed in the HybEZ Oven (Advanced Cell Diagnostics, Newark, CA, USA) and incubated at 40 °C for 2 h. Subsequently, signals were amplified using “Amp 1-FL”, “Amp 2-FL”, “Amp 3-FL”, and “Amp 4-FL-Alt A” reagents provided in the RNAscope Fluorescence Multiplex Kit. Reagents were applied to each section and sections were incubated in the HybEZ Oven for either 30 min (“Amp 1-FL”, “Amp 3-FL”) or 15 min (“Amp 2-FL”, “Amp 4-FL-Alt A”) at 40 °C. Slides were mounted with Pro-Long Gold Antifade (Invitrogen, Carlsbad, CA). Z-stack images were obtained using a 60× objective on a Nikon TE-2000E confocal microscope system.

The primary cell type expressing HIV-1 mRNA in the HIV-1 Tg rat was examined using two methods. First, RNAscope in situ hybridization images were compared to those of adjacent sections, which were processed with immunohistochemical staining for microglia (Iba1) and astrocytes (GFAP; methodology detailed below); a method utilized due to technological limitations. Second, RNAscope dual-labelling was utilized to more accurately assess the co-localization of expression for HIV-1 mRNA and specific cell type markers (i.e., microglia: Iba1 mRNA, astrocytes: GFAP mRNA) in primary microglia cultures from HIV-1 Tg rats (methodology detailed below) and brain tissue from HIV-1 Tg rats.

#### 2.3.1. Immunohistochemical Staining

In addition to RNAscope in situ hybridization, immunohistochemical staining was used to examine the localization of HIV-1 mRNA within different cell types (i.e., microglia, astrocytes) in brain tissue from the HIV-1 Tg rat. After perfusion (*n* = 6 male, HIV-1 Tg animals), brains were removed and post-fixed overnight in 4% cold paraformaldehyde. Brain tissue was sectioned into 50 µm thick coronal slices using a vibratome (PELCO easiSlicer^TM^, *TED PELLA, INC.* Redding, CA, USA). Brain sections were incubated with rabbit anti-Iba1 (ab178847, Abcam, Cambridge, United Kingdom) and rabbit anti-GFAP (ab7260, Abcam, Cambridge, UK) at 4 °C overnight. Subsequently, Alexa Fluor 594 goat anti-rabbit IgG (A11012, Invitrogen) was applied to brain sections and incubated for 4 h at room temperature. Z-stack images were obtained using a 60× objective on a Nikon TE-2000E confocal microscope system.

#### 2.3.2. Primary Microglia Isolation and Purification

To validate our observations, microglia cultures were harvested from HIV-1 Tg rats to examine the expression of HIV-1 mRNA.

First, mixed glia were isolated from the HIV-1 Tg rat on postnatal day (PD) 1 using a protocol that was modified from Moussaud et al. [47]. In brief, after the rats were deeply anesthetized, the brain was aseptically removed and the meninges were gently peeled off. A piece of the cortex was transferred to a small Petri dish containing 2 mL of DMEM/F12 medium, finely minced with a blade, transferred into a 15 mL centrifuge tube with DMEM/F12 medium and 0.5% Trypsin/EDTA, and incubated at 37 °C, 5% CO_2_ for 20 min. The digested cell suspension was gently pipetted 20 times using a 3.5 mL glass pipette and centrifuged at 1500 rpm for 4 min. The cell pellet was resuspended in DMEM/F12 medium with 10% FBS and the cells were seeded into a flask coated with poly-L-lysine. Cells were incubated at 37 °C, 5% CO_2_ overnight. Next, the DMEM/F12 medium with 10% FBS was replaced. Subsequently, the DMEM/F12 medium with 10% FBS was changed every three days.

Once the mixed glia reached 90% confluency (after 10–14 days), microglia were detached from the flask using an orbital shaker at 220 rpm for 1 h (37 °C). The DMEM/F12 medium with 10% FBS was changed no more than 72 h before isolation. The culture medium was aspirated and centrifuged at 1500 rpm for 4 min. Cells were resuspended and seeded into a cell culture place coated with poly-L-lysine. The purified primary microglia cells were maintained at 37 °C, 5% CO_2_ for 72 h before experimentation.

Isolation of primary microglia was verified by the immunohistochemical techniques described above (Antibodies: rabbit anti-Iba1, MOMA, and CD11b). Co-localization of HIV-1 viral mRNA with microglia was examined using the RNAscope in situ hybridization technique described above. Z-stack images were obtained with a Nikon TE-2000E confocal microscope system.

### 2.4. Experiment 2

EcoHIV Virus Construction and Preparation

The lentivirus of EcoHIV-NL4-3-EGFP, generously gifted from Dr. Potash of Icahn School of Medicine at Mount Sinai, was constructed from pNL4-3 in which the fragment of NL4-3 at nucleotide 6310 and NCA-WT at nucleotide 6229 was ligated to generate the chimeric virus. The virus stocks were prepared from the conditional medium after transfection of plasmid DNA into 293FT cells (Lipofectamine^TM^ 3000, Cat. No. L3000015, Invitrogen), and then concentrated using the lenti-x concentrator (Cat. No. 631231, Clontech Laboratories, Mountain View, CA, USA).

#### 2.4.1. Experiment 2.1: In Vitro

Primary microglia were isolated and purified using the protocol presented above. Following purification, primary microglia were infected with conditional medium of EcoHIV-EGFP from 293FT cell transfection. The co-localization of EcoHIV infection with microglia was examined using EGFP, which was integral to the lentivirus, and immunohistochemical techniques. Specifically, immunohistochemistry was utilized to visualize microglia using the rabbit anti-Iba1 antibody.

#### 2.4.2. Experiment 2.2: In Vivo, Seven Days Post-Infection

*EcoHIV-EGFP virus stereotaxic surgeries.* The goal of conducting EcoHIV-EGFP virus stereotaxic surgeries was twofold: (1) to determine whether EcoHIV successfully infects rat brain tissue, and (2) to determine whether EcoHIV expression in the brain is cell-type specific. Six adult Fisher 344/N rats were stereotaxically injected with the EcoHIV lentivirus (male, *n* = 4; female, *n* = 2). Specifically, rats were anesthetized with sevoflurane (Abbot Laboratories, North Chicago, IL, USA: catalog #035189). Next, rats were placed in the stereotaxic apparatus (Kopf Instruments, Tujunga, CA, USA: Model 900) and the scalp was exposed. The 0.40 mm diameter hole was bilaterally drilled into the skull in neuroanatomical locations relative to Bregma to infuse the viral vectors (1.5 μL EcoHIV NL4-3-EGFP, (1.04 × 10^6^ TU/mL) into the cortex (0.8 mm lateral, 1.2 mm anterior to Bregma, 2.5 mm depth). Viral vectors were infused at a rate of 0.2 μL/min through a 10 μL Hamilton syringe (catalog #1701).

*EcoHIV-EGFP virus retro-orbital injection.* Briefly, after completely anesthetized with isoflurane, the rat was positioned laterally with the injection-eye facing up. A 1 cc tuberculin syringe with a 26 G needle was slowly inserted into the medial canthus of the eye into the vessels behind the eye ball. Then, 20 µL of EcoHIV NL4-3-EGFP (1.04 × 10^6^ TU/mL) was gently injected into the retro-orbital vessels. The rat was placed back into the cage for recovery after inoculation.

*RNAscope in situ hybridization.* Methodology described above for RNAscope dual-labeling was utilized to examine the predominant cell type expressing HIV-1 mRNA seven days after EcoHIV was stereotaxically injected into the brain of the F344/N rat.

#### 2.4.3. Experiment 2.3: In Vivo, Eight Weeks Post-Infection

After confirming EcoHIV infection in the rat brain, a separate set of animals were used to assess neurocognitive dysfunction and the effect of EcoHIV infection on synaptic dysfunction, signaling pathways, and neuroinflammation. Twenty adult Fischer 344/N rats (male, *n* = 14; female, *n =* 6) were randomly assigned to receive stereotaxic injections of either EcoHIV (*N* = 10; female, *n* = 3; male, *n* = 7) or saline (i.e., control; *N* = 10; female, *n* = 3; male, *n* = 7).

*Neurocognitive assessments: Prepulse inhibition.* Prepulse inhibition (PPI) of the auditory startle response (ASR) and gap-prepulse inhibition (gap-PPI), tapping temporal processing, were conducted to determine whether EcoHIV infection produced neurocognitive impairments resembling those observed in HIV-1 seropositive individuals.

*Apparatus.* The startle chambers utilized to conduct habituation, cross-modal PPI, and gap-PPI have been previously reported [48]. In brief, an isolation cabinet (81 × 81 × 116 cm, Industrial Acoustic Company, INC., Bronx, NY, USA) enclosed a startle platform (SR-Lab Startle Reflex System, San Diego Instruments, Inc., San Diego, CA, USA) to provide sound attenuation (30 db(A)) relative to the external environment. A SR-Lab system high-frequency loudspeaker (model#40-1278B, Radio Shack, Fort Worth, TX, USA), mounted 30 cm above the Plexiglas animal test cylinder, was used to present continuous background noise (22 dB(A)), auditory prepulse stimuli (85 db(A), duration: 20 ms), and the startle stimulus (100 db(A), duration: 20 ms). A 22 lux white LED light was utilized to present visual prepulse stimuli (duration: 20 ms). A piezoelectric accelerometer converted the deflection from the test cylinder into analog signals. These signals were recorded (12 bit A to D) at a rate of 2000 samples/s. Two individual startle apparatuses were utilized throughout the duration of the experimentation.

*Procedure.* Temporal processing was assessed using cross-modal PPI and auditory gap-PPI in EcoHIV and control animals approximately two weeks after stereotaxic injections.

Following a single session of habituation (methodology reported by Moran et al. [32]), cross-modal PPI and auditory gap-PPI were conducted in a sequential manner. Each session began with a 5-min acclimation period under continuous 70 dB(A) white background noise followed by 6 pulse-only ASR trials with a fixed 10 ms intertrial interval (ITI). For cross-modal PPI, a total of 72 trials were presented including an equal number of auditory and visual prepulse trials. The prestimulus modality (i.e., auditory or visual) was arranged using an ABBA counterbalanced order of presentation. For auditory gap-PPI, a 20-ms gap in background noise served as a discrete prestimulus during 36 testing trials. Trials for both cross-modal PPI and auditory gap-PPI had interstimulus intervals (ISIs) of 0, 30, 50, 100, 200, or 4000 ms, which were presented in 6-trial blocks interdigitated using a Latin Square experimental design. ISIs of 0 and 4000 ms served as control trials to provide a reference ASR within the testing session. A variable (15–25 s) ITI was employed for all testing trials. Mean peak ASR amplitude values were collected for analysis.

#### 2.4.4. Neurocognitive Assessments: Locomotor Activity

*Apparatus.* Square (40 cm × 40 cm) activity monitors (Hamilton Kinder, San Diego Instruments, San Diego, CA, USA) were converted into round (~40 cm diameter) compartments using perspex inserts. Interruptions of infrared photocells (32 emitter/detector pairs) were utilized to detect free movement.

*Procedure.* Locomotor activity was conducted on three consecutive days [49] approximately seven weeks after stereotaxic injections of either EcoHIV or saline using a sixty-minute test session. Test sessions were conducted between 7:00 and 12:00 h (EST) in an isolated room under dim lighting conditions (<10 lux).

*RNAscope in situ hybridization.* Viral infection and replication were verified by labeling HIV-1 mRNA and HIV-1 DNA using the RNAscope in situ hybridization methods detailed above. NogoA signaling in EcoHIV (*N* = 4, female, *n* = 1, male, *n* = 3) and control (*N* = 3, female, *n* = 1, male, *n* = 2) animals was also examined using RNAscope in situ hybridization.

*Synaptic dysfunction.* Rats were anesthetized with sevoflurane (Abbot Laboratories, North Chicago, IL, USA) and transcardially perfused with 4% paraformaldehyde perfusion. Next, a ballistic labeling methodology was used to visualize pyramidal neurons from layers II–III of the medial prefrontal cortex (mPFC; 3.7 mm to 2.2 mm anterior to Bregma) [50] and medium spiny neurons from the nucleus accumbens (NAc; approximately 2.8 mm anterior to Bregma) [50]. Methodological details describing ballistic cartridge preparation and methodology of DiOlistic labeling are available in Li et al. [51].

Z-stack images were obtained on three pyramidal neurons and three MSNs from each animal (EcoHIV: *N* = 10, female, *n* = 3; male, *n* = 7; control: *N* = 10, female, *n* = 3; male, *n* = 7) using a Nikon TE-2000E confocal microscope system. According to the selection criteria (i.e., low background/dye clusters, minimal diffusion of the DiI dye into the extracellular space, and continuous dendritic staining), one pyramidal neuron and one MSN from each animal were chosen for analysis. Following selection, Neurolucida 360 (MicroBrightfield, Williston, VT, USA), a sophisticated neuronal reconstruction software, was utilized to examine neuronal morphology and dendritic spine morphology. Specifically, dendritic spine morphology was evaluated using parameters of head diameter (µm) and neck diameter (µm).

*Signaling pathways.* Key elements of the Nogo-A signaling pathway, which is involved in synaptic plasticity in the CNS [52], were examined using tissue fluorescence immunostaining techniques (methodology detailed above; EcoHIV: *N* = 6, female, *n* = 2; male, *n* = 4; control: *N* = 6, female, *n* = 2, male, *n* = 4). Specifically, brain sections were incubated with rabbit anti-PirB (E-AB-15732, Elabscience, Houston, TX, USA) at 4 °C overnight, which potentially inhibited the axonal regeneration and synaptic plasticity following CNS injury [53]; mouse anti-NgR3 (sc-515400, Santa Cruz Biotech, Dallas, TX, USA), whose function is poorly characterized; or mouse anti-Rho A (sc-418, Santa Cruz Biotech, Dallas, TX, USA), whose inhibition is critical to spine maintenance (for review [54]). Then, brain sections were incubated with goat anti-rabbit Alexa Fluor 594 (A11012, Invitrogen, Carlsbad, CA, USA), or donkey anti-mouse Alexa Fluor 594 (A21203, Invitrogen, Carlsbad, CA, USA).

*Neuroinflammatory markers.* The total RNA was isolated from 20 mg of brain tissue (EcoHIV: *N* = 4, female, *n* = 1 male, *n* = 3; control: *N* = 4, female, *n* = 1, male, *n* = 3) using the RNeasy Mini Kit (Cat. No. 74104, QIAGEN, Hilden, German). One µg of total RNA was used for cDNA synthesis through the Cloned AMV first-strand cDNA Synthesis Kit (Invitrogen, Carlsbad, CA, USA). The first-strand cDNA synthesis reaction mixture included: 1 µg total RNA sample, 5x cDNA synthesis buffer, 2 μL of 10 mM dNTP mix, 1 μL random-hexamer primer, 1 μL of 0.1 M DTT, 1 µL cloned AMV RT, 1 μL RNaseOUT, and 1 µL DEPC-treated H_2_O. The first-strand cDNA synthesis conditions were: 65 °C (5 min), 25 °C (10 min), 50 °C (50 min), and 85 °C (5 min). Then, neuroinflammation associated cytokines (TNF-α, IL-1β, NF-κB, IL-6) were detected using real-time PCR with a SsoAdvanced Universal SYBR Green Supermix Kit (BIO-RAD). The reaction included 1 μL of cDNA product (100 ng), 10 μL of 2× SsoAdvanced universal SYBR Green supermix, 1 μL of forward and reverse primers (250 nM each), and 7 μL of DEPC-treated H_2_O. The PCR conditions were: 95 °C for 30 s and 40 cycles of 95 °C for 15 s and 59 °C for30 s using the DNA Engine Opticon 2 system (Bio-Rad, Hercules, CA, USA). Supplementary Table S1 shows the detail of all primers, and the internal control was β-Actin. The 2^−ΔΔCt^ method was performed to examine relative changes of each neuroinflammatory marker.

### 2.5. Statistical Analyses

Regression and analysis of variance (ANOVA) techniques were utilized to statistically analyze the data (SAS/STAT Software 9.4, SAS Institute, Inc., Cary, NC, USA; SPSS Statistics 26, IBM Corp., Somer, NY, USA; GraphPad Software, Inc., La Jolla, CA, USA). Figures were drawn using GraphPad Prism 5 (GraphPad Software Inc., La Jolla, CA, USA. An alpha value of *p* ≤ 0.05 was considered statistically significance.

The regional distribution of HIV-1 mRNA in the HIV-1 Tg rat was analyzed using repeated-measures ANOVA, whereby brain region (i.e., mPFC, NAc, and hippocampus (HIP)) served as the within-subjects factors.

Body weight in EcoHIV animals was also analyzed using repeated measures ANOVA. Age served as the within-subjects factor, while genotype (i.e., EcoHIV vs. control) and biological sex (i.e., female vs. male) served as between-subjects factors. Regression analyses were conducted to evaluate neurocognitive deficits (i.e., temporal processing, long-term episodic memory) in EcoHIV animals.

Dendritic branching complexity, evaluated by the centrifugal branch ordering method and dendritic arbor complexity, assessed using the classical Sholl analysis [55], were analyzed using regression analyses. Furthermore, morphological parameters of dendritic spines including head diameter and neck diameter were analyzed using a generalized linear mixed effects model with a Poisson distribution with an unstructured covariance pattern. The number of dendritic spines within each bin served as the dependent variable, and bin served as a within-subjects factor. A univariate ANOVA was conducted to evaluate the EcoHIV-induced changes in the expression of NogoA, NgR3, PirB, and RhoA. Biological sex was included as a covariate in the analysis of the Nogo A-NgR3/PirB-RhoA signaling pathway.

## 3. Results

### 3.1. Experiment 1: HIV-1 Transgenic Rats

#### 3.1.1. A Restricted and Region-Specific Distribution of HIV-1 mRNA Was Detected in HIV-1 Tg Rats

**Figure 2:**
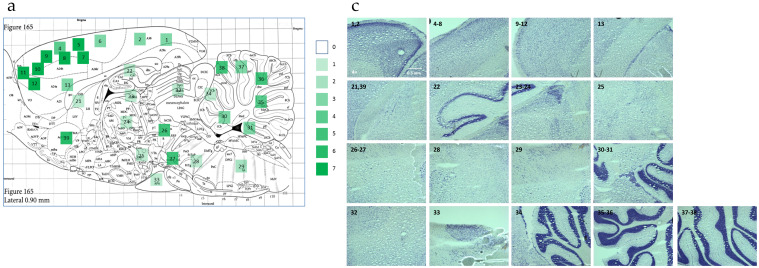

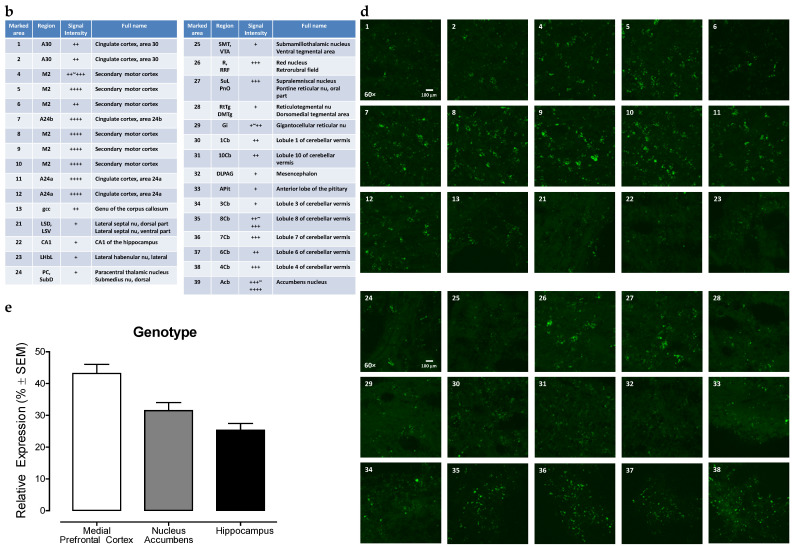
Restricted and region-specific HIV-1 distribution in the HIV-1 Tg rat brain assessed using RNAscope in situ hybridization. (**a**) The sagittal diagram illustrates the brain regions with HIV-1 expression, and relative intensity, in the HIV-1 Tg rat brain. (**b**) All of the HIV-1 expression areas in HIV-1 Tg rat are listed with the intensity, assessed on a scale from 0 (no HIV-1 expression) to 7 (high HIV-1 expression). The schematic map of sagittal section (lateral 0.90 mm) is cited from *Paxions and Watson’s The Rat brain in Stereotaxic Coordinates* (7th Edition) [50]. (**c**) Representative images (4×) of sagittal brain sections using Nissl staining in brain regions with HIV-1 expression are presented. The regions with detectable HIV-1 expression are labeled on the sagittal diagram (Figure 2a). (**d**) Representative confocal images (60×) of HIV-1 expression in different regions of HIV-1 Tg rat brain. Green fluorescence represents the HIV-1 mRNA expression labeled using RNAscope in situ hybridization. (**e**) Relative expression of region-specific HIV-1 mRNA in mPFC, NAc, HIP.

HIV-1 Tg rats showed a restricted, region-specific distribution of HIV-1 mRNA, evidenced by RNAscope in situ hybridization. HIV-1 expression and intensity were categorized across multiple brain regions using an ordinal scale, where 0 reflected no HIV-1 mRNA expression and 7 represented high HIV-1 mRNA expression (Figure 2a,b). Phase-contrast Nissl staining images show the brain regions where HIV-1 mRNA expression was detected (Figure 2c). The green fluorescence signals observed in confocal images (Figure 2d) revealed the restricted HIV-1 mRNA expression (single dot expression pattern) of each marked region labeled using the RNAscope in situ hybridization assay.

The relative frequency of green fluorescent dots was quantified in three brain regions associated with higher-order cognitive processes including the mPFC [56], NAc [57,58], and HIP [59]. The most abundant HIV-1 mRNA expression was observed in the mPFC (Figure 2e). Relative to the mPFC, the NAc and HIP exhibited lower levels of HIV-1 mRNA expression. A repeated-measures ANOVA confirmed our observations, revealing a statistically significant effect of brain region [*F*(2,4) = 9.2, *p* ≤ 0.03, η_p_^2^ = 0.821] and a main effect of biological sex [*F*(1,2) = 178.5, *p* ≤ 0.001, η_p_^2^ = 0.989]. Notably, other brain regions including the substantia nigra (SN) and cerebellum also exhibited elevated HIV-1 mRNA expression.

#### 3.1.2. Cell-Type Specific HIV-1 mRNA Expression Was Observed in HIV-1 Tg Rat

**Figure 3:**
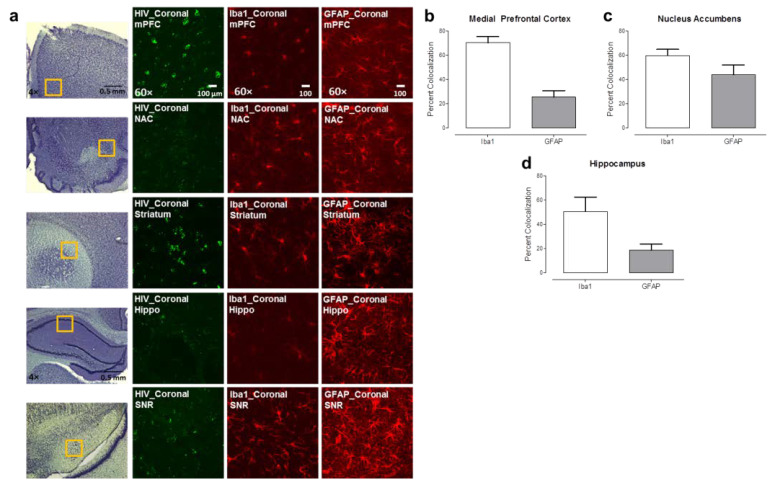
HIV-1 mRNA expression in the mPFC, NAc, HIP, and SN of the HIV-1 Tg rat. (**a**) The yellow frame indicates the selected region, determined via Nissl staining, for confocal imaging. Green fluorescence signal represents HIV-1 mRNA expression. Red fluorescence signal shows the cell type (i.e., microglia, astrocytes) based on IHC staining for either Iba1 or GFAP, respectively. (**b**–**d**) Statistical analysis of dual labeling of HIV-1 with each type of cell marker in mPFC, NAc, and HIP using RNAscope in situ hybridization. The overall percent co-localization and number of HIV-1 mRNA copies in different regions and cell types is illustrated.

Subsequently, we investigated whether HIV-1 mRNA expression was cell-type specific by conducting RNAscope in situ hybridization and immunohistochemical staining for microglia (Iba1) and astrocytes (GFAP) on adjacent brain sections. The majority of green fluorescent dots, each reflecting a single copy of HIV-1 mRNA expression, were located inside of microglia, which were identified based on cell morphology (Figure 3a). Unfortunately, due to technological limits, it was not possible to combine the RNAscope in situ hybridization assay with immunohistochemical staining in the same sections.

In response to these limitations, we utilized the RNAscope dual-labeling assay, affording an opportunity to combine cell-type specific probes (Iba1 and GFAP) with the HIV-1 probe. The percent of co-localization between cell-type specific mRNA expression and HIV-1 mRNA expression were quantified in the mPFC, NAc, and HIP regions in HIV-1 Tg rat brain (Figure 3b–d). HIV-1 mRNA expression primarily co-localizes with microglia in the mPFC, NAc, and HIP. Thus, two complementary techniques including immunohistochemical staining and the RNAscope in situ dual-labeling assay, provide strong, independent evidence for the expression of HIV-1 mRNA in microglia.

#### 3.1.3. Purified Microglia Validated Cell-Type Specific HIV-1 Expression

**Figure 4:**
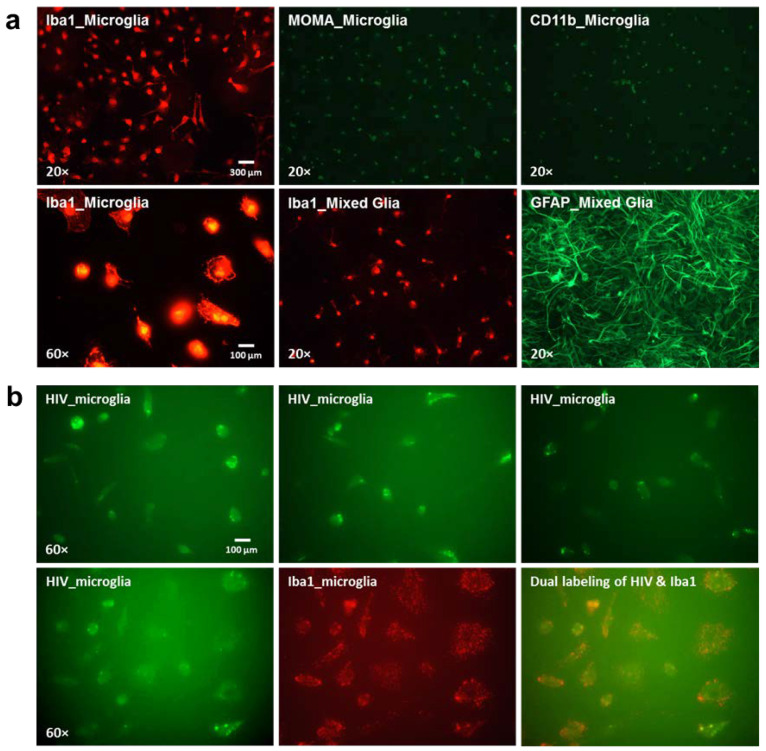
Primary microglia isolation, purification, and validation in HIV-1 Tg rat. (**a**) Representative fluorescence images of the purified microglia cells, which were verified by microglia cell markers: Iba1 (red), MOMA (green), and CD11b (green) at three days after purification through IHC staining. GFAP was used as an indicator of astrocytes in the mixed glia. (**b**) Representative fluorescence images of the purified microglia cells were verified by dual-labelling of Iba1 and HIV probe through RNAscope in situ hybridization. The three images in the upper panel represented the HIV-1 mRNA expression from three individual experiments by RNAscope in situ hybridization. The lower panel shows the co-localization of HIV-1 mRNA with purified microglia cells in vitro.

To validate our observations in the HIV-1 Tg rat brain, we isolated and purified microglia from mixed glia cells in HIV-1 Tg rats. Several cell markers specific to microglia (Iba1, MOMA, and CD11b) were chosen for immunofluorescence staining to verify the purity of our microglia. Purified microglia (Figure 4a) showed a strong fluorescence signal for all cell markers (Iba1, red; MOMA, green; CD11b, green), indicating reliable purity. Astrocytes (Figure 4a) were labeled with GFAP (green) within the mixed glia culture. Dual-labelling of HIV-1 and Iba1 mRNA using RNAscope in situ hybridization (Figure 4b) revealed significant co-localization between HIV-1 and Iba1-positive cells, supporting the role of microglia in brain cells of HIV-1 Tg rats both in vivo and in vitro.

### 3.2. Experiment 2: EcoHIV Infection Model in Rats

#### 3.2.1. Experiment 2.1: In Vitro

**Figure 5:**
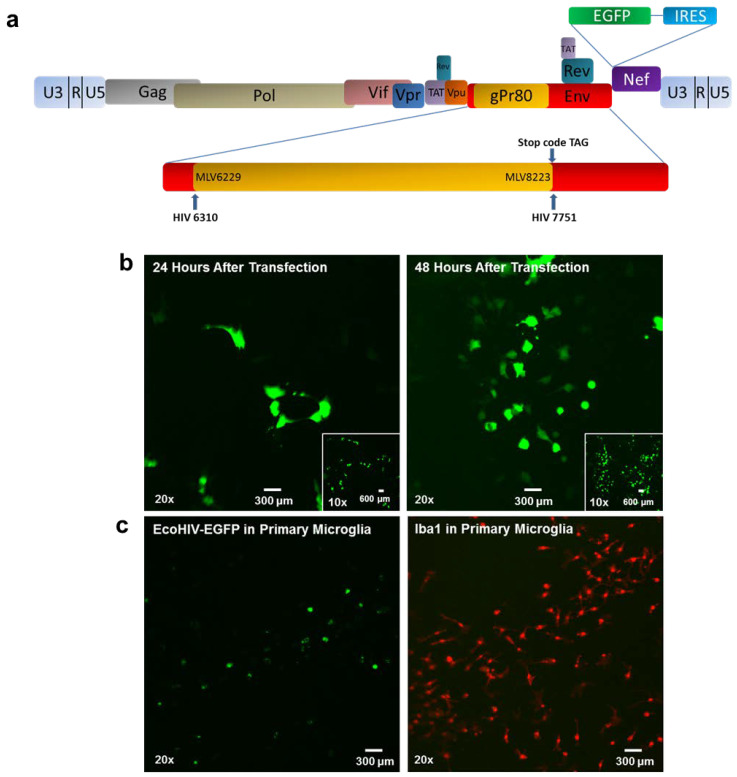
Structure of EcoHIV. (**a**) The schematic map of EcoHIV-NL4-3-EGFP. The HIV-1 env was replaced by the MLV ecotropic envelope gp80 with the stop codon. (**b**) The EcoHIV-EGFP plasmid was transfected into 293FT cell for virus packaging. (**c**) Twenty-four hours after viral infection, rat primary microglia were immunostained with the Iba1 antibody.

*EcoHIV successfully infects rat primary microglia in vitro.* To develop an innovative biological system to model key aspects of HIV-1 infection, we constructed a lentivirus of EcoHIV-EGFP to be applied to healthy rat brain cells in vitro and in vivo. Figure 5a shows the construction of EcoHIV-EGFP on a backbone of clade B NL4-3 with the replacement of the gp120 gene (the coding region of HIV-1 surface envelope glycoprotein) by the ecotropic MLV gp80 gene for entry into cells through CAT-1. The plasmid DNA of EcoHIV-EGFP was transfected into 293FT cells to package the lentivirus (Figure 5b). The conditional medium that included the EcoHIV-EGFP lentivirus was collected and co-cultured with primary microglia isolated from F344/N rat for 24 h (Figure 5c). EcoHIV-EGFP was well expressed in microglia (i.e., Iba1 positive cells validated by Iba1 immunostaining), indicating rat primary microglia can be infected by EcoHIV in vitro.

#### 3.2.2. Experiment 2.2: In Vivo, Seven Days Post-Infection

**Figure 6:**
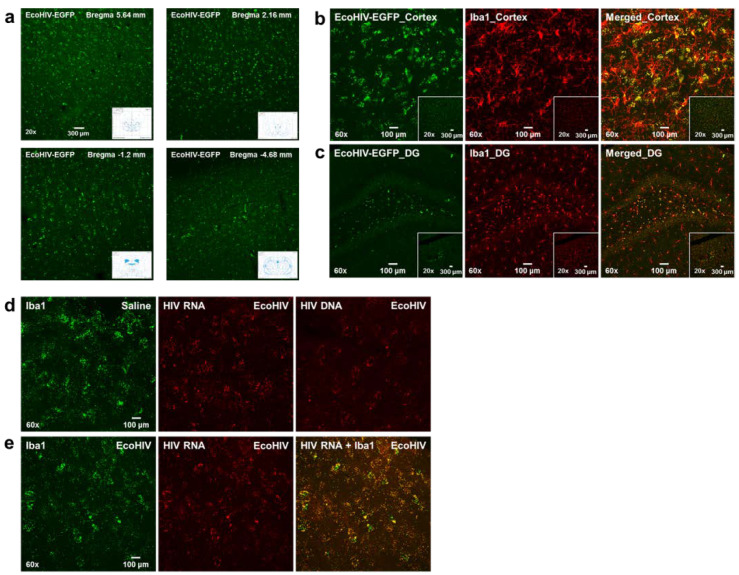
EcoHIV-EGFP infected cells in rat brain. (**a**) Four representative confocal images (from different coronal sections; bregma: 5.60 mm, 2.16 mm, −1.2 mm, −4.68 mm) of RNAscope in situ hybridization show infected cells in rat brain at seven days after the stereotaxic injection. (**b**,**c**) Representative images of co-localization of Iba1 immunostaining cortex or hippocampal dentate gyrus regions with EcoHIV-EGFP infected cells seven days after injection. (**d**,**e**) EcoHIV mRNA or DNA, microglia marker Iba1 expression and dual labeling of EcoHIV mRNA with microglia marker, Iba1, in cortex by RNAscope in situ hybridization eight weeks after injection.

*Significant infection was observed in microglia seven days after stereotaxic injection of EcoHIV into the rat brain.* The conditional medium, including the lentivirus of EcoHIV-EGFP from the infected 293FT cells, was concentrated, tittered, and stereotaxically injected into the cortex of F344/N rats (*N* = 6; female, *n* = 2, male, *n* = 4). Seven days after infection, animals were sacrificed and images were taken from coronal brain slices, spanning Bregma 5.64 mm to Bregma −4.68 mm, revealing significant EcoHIV-EGFP expression throughout the brain (Figure 6a, and Supplementary Figure S1). In both the cortex and the hippocampal dentate gyrus, Iba1 immunostaining co-localized with EcoHIV-EGFP fluorescence signals, providing strong evidence that microglia are potentially the predominant cell type harboring EcoHIV virus in the brain (Figure 6b,c). Additionally, retro-orbital injections of EcoHIV-EGFP into F344/N rats (*N* = 4; female, *n* = 2, male, *n* = 2) also elicited high expression of EcoHIV-EGFP in both the cortex and hippocampal dentate gyrus after seven days of infection (see Supplementary Figure S2). The distribution pattern of viral mRNA routed by retro-orbital injection was similar to stereotaxic injection of EcoHIV-EGFP, and microglia were still the predominant cell type for EcoHIV mRNA expression.

#### 3.2.3. Experiment 2.3: In Vivo, Eight Weeks Post-Infection

EcoHIV infection forms viral reservoirs in microglia eight weeks after stereotaxic injections of EcoHIV into the rat brain. The persistence of EcoHIV infection in the brain was assessed using RNAscope in situ hybridization to determine (1) if both EcoHIV mRNA and DNA were expressed and (2) the cell type harboring HIV-1 DNA in the brain. High levels of HIV-1 mRNA and DNA were observed in the prefrontal cortex as well as other brain regions (Figure 6d,e). Furthermore, dual-labeling of brain tissue from EcoHIV-infected rats revealed co-localization of HIV mRNA with microglia (i.e., Iba1 positive cells). Most critically, microglia serve as a viral reservoir for HIV-1, as evidenced by their ability to harbor EcoHIV DNA in brain tissue (Figure 6d). Collectively, the results validate observations in the HIV-1 Tg rat, providing strong evidence for the role of microglia in HIV infection.

**Figure 7:**
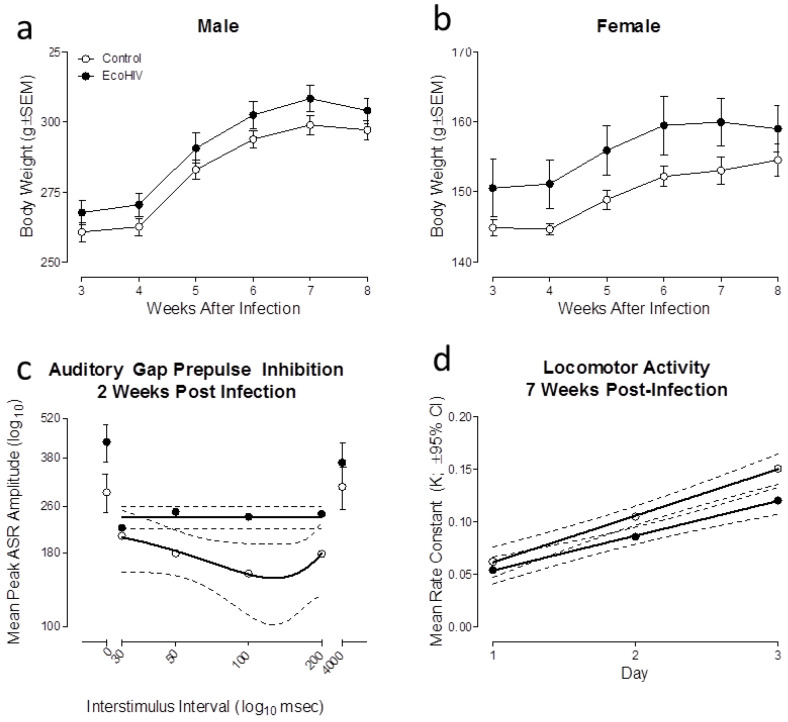
EcoHIV infection induced prominent neurocognitive deficits in temporal processing and long-term episodic memory. (**a**,**b**) EcoHIV and control animals, independent of biological sex, exhibited steady growth throughout the duration of the experiment. (**c**) Auditory gap prepulse inhibition was conducted two weeks after stereotaxic injections of either EcoHIV or saline. EcoHIV infection induced prominent alterations in temporal processing evidenced by the relative insensitivity to the manipulation of interstimulus interval relative to control rats. (**d**) Three consecutive test sessions in locomotor activity were conducted seven weeks after stereotaxic injections. Alterations in the mean rate constant in EcoHIV rats supports prominent deficits in long-term episodic memory relative to control rats.

EcoHIV rats, independent of biological sex, exhibited prominent neurocognitive dysfunction in long-term episodic memory and temporal processing. The functional health of EcoHIV and control animals was evidenced using body weight as an assessment of somatic growth. (Figure 7a,b). Both EcoHIV and control animals gained weight throughout the duration of the experimentation (main effect: Week, [*F*(5, 80) = 287.9, *p* ≤ 0.001, η_p_^2^ = 0.947]). The rate of growth was dependent upon biological sex (week x sex interaction, [*F*(5, 80) = 108.3, *p* ≤ 0.001, η_p_^2^ = 0.871]). There was no evidence, however, of any effect of EcoHIV infection on somatic growth (*p* > 0.05).

Temporal processing, a potential neurobehavioral mechanism underlying alterations in higher-order cognitive functions [60], was assessed using auditory gap-PPI. EcoHIV rats showed a relative insensitivity to the manipulation of ISI compared with the control rats (Figure 7c). Regression analyses confirmed these observations, evidenced by differences in the functional relationship describing the inhibition curve during the gap-PPI testing trials (i.e., 30–200 ms ISI). Specifically, a second-order polynomial provided a well-described fit for control animals (*R*^2^ ≥ 0.98); maximal inhibition was observed at the 100 ms ISI. A horizontal line, however, afforded the best fit for EcoHIV animals, supporting insensitivity to the manipulation of ISI. Therefore, the results support a prominent alteration in temporal processing in EcoHIV animals, an alteration that occurs early during the course of viral protein exposure.

Long-term episodic memory, which is commonly altered in HIV-1 seropositive individuals [61], can be assessed using measures of novelty and habituation [62,63,64]. EcoHIV animals exhibited a prominent alteration in long-term episodic memory, evidenced by a differential development of intrasession habituation, relative to control animals. During each locomotor activity test session, a one-phase decay afforded a well fit for the mean number of photocell interruptions during locomotor habituation trials in both the EcoHIV and control animals (*R*^2^s ≥ 0.96). The rate of intrasession habituation (i.e., *K*) during each test session, however, was dependent upon genotype (Figure 7d). In particular, a first-order polynomial afforded the best fit for the rate of intrasession habituation in both the EcoHIV and control animals (*R*^2^s ≥ 0.99). Statistically significant differences in the slope (i.e., β_1_), however, were observed (*F*(1,2) = 91.0, *p* ≤ 0.01), supporting a slower development of intrasession habituation in EcoHIV animals. Thus, EcoHIV animals exhibited prominent neurocognitive impairments, characterized by alterations in temporal processing and long-term episodic memory.

#### 3.2.4. Prominent Alterations in Neuronal Morphology and Synaptic Function Were Observed in EcoHIV Animals

A ballistic labeling technique and sophisticated neuronal reconstruction software were utilized to examine dendritic spine and neuronal morphology in pyramidal neurons from layers II–III of the mPFC, a brain region associated with higher-order cognitive processes.

**Figure 8:**
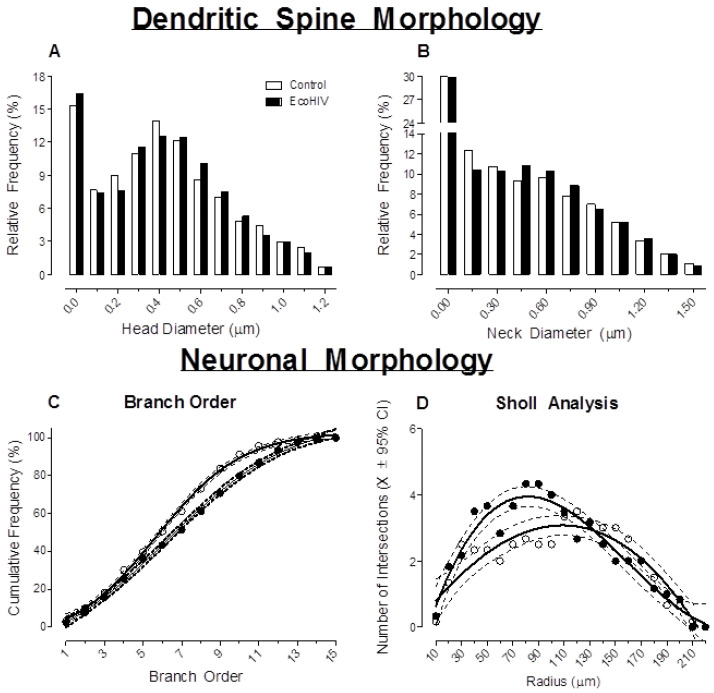
EcoHIV infected animals displayed prominent alterations in dendritic spine and neuronal morphology in pyramidal neurons from layers II–III in medial prefrontal cortex. (**A**,**B**) Rats infected with EcoHIV exhibited a profound morphological shift accompanying an increased relative frequency of dendritic spines (increased head diameter and neck diameter); a morphological shift consistent with a ‘stubby’ dendritic spine phenotype. (**C**,**D**) EcoHIV animals exhibited prominent alterations in neuronal arbor complexity and dendritic branching, evidenced by alterations in dendritic branch order and Sholl analysis, respectively.

EcoHIV infection induced prominent alterations in the morphological parameters (i.e., head diameter, neck diameter) of dendritic spines in pyramidal neurons (Figure 8A,B). Specifically, EcoHIV infected rats showed a population shift toward dendritic spines with increased head diameter (treatment x bin interaction, *F*(12,96) = 1.9, *p* ≤ 0.05) and increased neck diameter (treatment x bin interaction, pyramidal neurons: *F*(10,80) = 2.0, *p* ≤ 0.05) relative to control animals; morphological parameters supporting a population shift towards a ‘stubby’ phenotype. Pyramidal neuronal morphology was also altered by EcoHIV infection, evidenced by profound changes in dendritic branching complexity (Figure 8C) and dendritic arbor complexity (Figure 8D). Dendritic branching complexity was examined using a method of assigning a branch order based on the number of segments traversed. EcoHIV infection increased the frequency of higher-order dendritic branches relative to control animals (regression fit: sigmoidal dose-response, *R*^2^s ≥ 0.99; Treatment differences in the parameters of the function: *F*(4,22) = 34.7, *p* ≤ 0.001). Furthermore, dendritic arbor complexity was assessed using the classical Sholl analysis [48]. Increased dendritic arbor complexity was observed proximal to the soma in EcoHIV infected animals relative to the controls (regression fit: third order polynomial, *R*^2^s ≥ 0.86; treatment differences in the parameters of the function: *F*(4,36) = 7.3, *p* ≤ 0.001). In combination, the results suggest that infection with EcoHIV may have interfered with synaptogenesis, a process of dendritic and synaptic pruning that occurs in the prefrontal cortex during adolescence and young adulthood [65,66]. Furthermore, it is notable that the factor of biological sex likely interacts with EcoHIV infection, evidenced by a statistically significant treatment x sex x bin interaction (*p* ≤ 0.05) for both head diameter and neck diameter. Given these observations, there is a critical need to conduct additional studies with increased sample size to more fully elucidate the role of biological sex.

#### 3.2.5. EcoHIV Activated the NogoA-NgR3/PirB-RhoA Signal Pathways, Which May Mechanistically Underlie the Prominent Alterations in Neuronal Morphology and Synaptic Function

**Figure 9:**
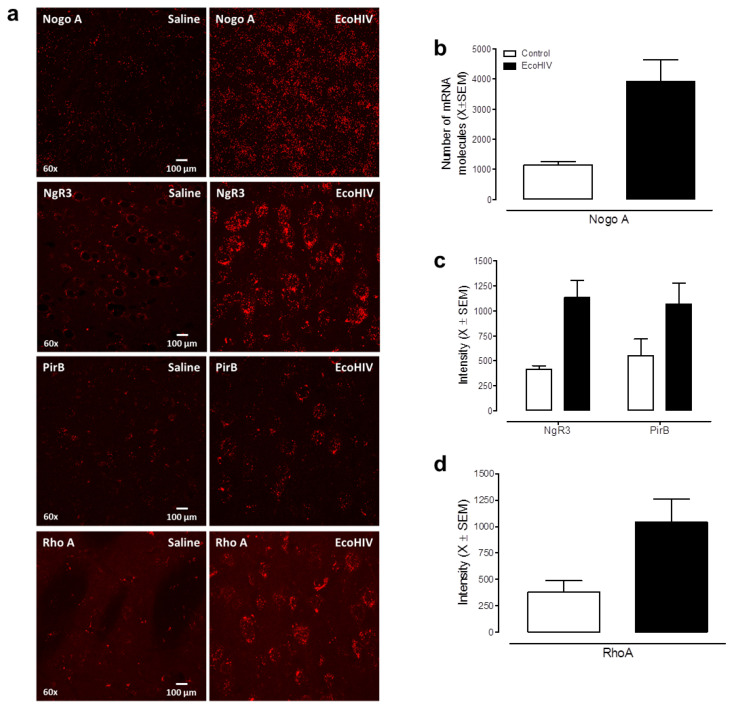
EcoHIV infection activated the NogoA-NgR3/PirB-RhoA signal pathways. (**a**) Eight weeks after EcoHIV infection, NogoA mRNA was detected by RNAscope in situ hybridization in the cortex; NgR3, PirB, and RhoA expression utilized immunofluorescence staining in EcoHIV infected and control rats. (**b**–**d**) Overall, EcoHIV infected rats showed a significant increase in NogoA, NgR3, and RhoA expression in the brain compared to the control group.

The NogoA-NgR3/PirB-RhoA signaling pathway is involved in the inhibition of axon growth and destabilization of synapses [67]. Eight weeks after EcoHIV infection, the NogoA-mediated signaling pathway was activated, evidenced by the upregulation of NogoA (*F*(1,6) = 9.4, *p* ≤ 0.04, η_p_^2^ = 0.702), NgR3 (*F*(1,12) = 16.3, *p* ≤ 0.003, η_p_^2^ = 0.645), and RhoA (*F*(1,12) = 8.1, *p* ≤ 0.02, η_p_^2^ = 0.475) expression relative to the control animals (Figure 9a–d). Alterations in the regulation of PirB were not statistically significant (*p* > 0.05). Given the well-recognized role of the NogoA-NgR3/PirB-RhoA signaling pathway in synaptic function, future studies should investigate whether this pathway mechanistically underlies the prominent alterations in neuronal morphology and synaptic function following EcoHIV infection.

#### 3.2.6. Significant Differences in Neuroinflammation between EcoHIV Infected and Control Rats

**Figure 10:**
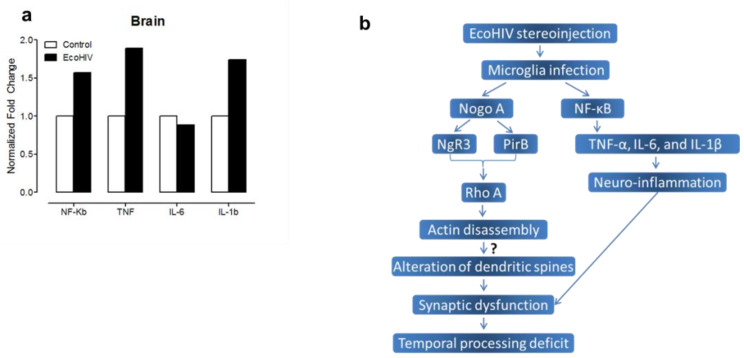
Neuroinflammation in the brains of EcoHIV infected and control rats. (**a**) Eight weeks after EcoHIV infection, four neuroinflammatory markers including NF-κB, TNF-α, IL-6, and IL-1β were assessed in the cortex of EcoHIV infected and control animals. Data are presented as normalized fold changes. (**b**) A schematic view of the potential mechanism by which the NogoA-NgR3/PirB-RhoA signaling pathway and/or neuroinflammation underlie synaptic dysfunction.

In addition to synaptic dysfunction, neuroinflammation has been implicated as another mechanism underlying HAND. To further establish the utility of the EcoHIV rat as a valid biological system to model HIV infection, four putative neuroinflammatory markers (i.e., NF-κB, TNF-α, IL-6, and IL-1β) were measured in the brain and quantified using the 2^−ΔΔCt^ method. A 1.57-fold increase in NF-κB was detected in the brain tissue of EcoHIV infected animals eight weeks after viral infusion. Furthermore, increased expression levels of TNFα (1.88 fold), IL-1β (1.73 fold), and IL-6 (0.88 fold) in the brain were also observed in EcoHIV infected animals (Figure 10a).

## 4. Discussion

In both the HIV-1 Tg rat and EcoHIV rat, microglia are the primary cell-type expressing HIV-1 mRNA supporting a HIV-1 viral reservoir in the brain; a reservoir that may underlie the persistence of HAND despite cART. In the HIV-1 Tg rat, a restricted, region-specific distribution of HIV-1 mRNA was observed, with the highest level of expression in the mPFC, NAc, SN, cerebellum, and HIP. In EcoHIV rats, an extension of the EcoHIV infection in mice, HIV-1 mRNA was expressed as early as seven days after infection and was localized in microglia. A critical test of the EcoHIV rat across eight weeks revealed the persistence of EcoHIV infection in the brain. Neurocognitive and neuroanatomical assessments in EcoHIV rats support the utility of the EcoHIV rat as a biological system to model HAND in the presence of active HIV infection. Collectively, EcoHIV infection in rat replicates observations in the HIV-1 Tg rat, enhancing our understanding of HIV-1 in the brain and offering a novel biological system to also model HAND and associated comorbidities (i.e., drug abuse).

With regard to HAND, a common consequence of HIV-1 in the post-cART era, the restricted, region-specific distribution of HIV-1 mRNA in the brain of HIV-1 Tg animals was notable. Across the 39 brain regions that were examined (see Figure 2a,b), the highest levels of mRNA expression were observed in the mPFC, NAc, SN, cerebellum, and HIP; brain regions associated with neurocognitive and neurobehavioral functions commonly altered in HAND. Specifically, the mPFC is associated with multiple neurocognitive functions including temporal processing [68], attention [69], and executive functions [70]; functions that are commonly altered in HIV-1 seropositive individuals [71] and the HIV-1 Tg rat [33,48]. Furthermore, neurocognitive functions related to the hippocampus including memory are altered by HIV-1 viral proteins [31,72]. Neurobehavioral alterations associated with the NAc including motivational dysregulation are also commonly observed in HIV-1 seropositive individuals [73] and have been translationally modeled in the HIV-1 Tg rat [74]. Taken together, the highest levels of HIV-1 mRNA expression were observed in brain regions commonly associated with neurocognitive and neurobehavioral functions altered by HIV-1 viral proteins, observations that support the HIV-1 Tg rat as a valid biological model for studying the key aspects of HAND in the post-cART era.

Furthermore, brain regions exhibiting high HIV-1 mRNA expression also correspond with neuroanatomical alterations previously observed in the HIV-1 Tg rat. Broadly, the HIV-1 Tg rat exhibits synaptic dysfunction [33,36], neurotransmitter system alterations [37,75], and neuroinflammation [40]; deficits that have been implicated in the pathogenesis of HAND. More specifically, profound synaptic dysfunction has been observed in both pyramidal neurons from layers II–III of the mPFC [33] and medium spiny neurons from the NAc [36,76], as evidenced by alterations in dendritic branching complexity and synaptic connectivity in HIV-1 Tg rats relative to the controls. Alterations in intrinsic excitability have also been observed in CA1 pyramidal neurons from the HIP [77]. Furthermore, prominent alterations in dopaminergic and serotonergic function have been observed in both the mPFC and NAc [37]. Considering the relatively high expression of HIV-1 mRNA observed in the mPFC, NAc, and HIP, the existence and persistence of viral reservoirs may lead to synaptic dysfunction and neurotransmitter system alterations.

Most critically, the utilization of dual-labeling revealed prominent co-localization of HIV-1 mRNA and microglia in vivo and in vitro, supporting microglia as one of the major cell types in which HIV-1 is expressed. Our data indicate that more than 50% of Iba1-positive cells (i.e., microglia) harbor HIV-1 mRNA in the tissues of HIV-1 Tg rats (Figure 3b–d) and in vitro purified primary microglia. Additionally, previous studies have reported prominent alterations in both the number [34] and morphology [78] of microglia in the HIV-1 Tg rats. The noninfectious nature of the HIV-1 Tg rats, however, precludes HIV-1 viral replication, necessitating the extension of a novel biological system (i.e., EcoHIV) to rats for the assessment of the functional role of microglia in HIV-1 infection. Persistent EcoHIV mRNA and DNA were detected in microglia eight weeks after viral infection, supporting microglia as a viral reservoir for HIV-1 during active infection, results that are consistent with previous clinical [5,17,18] and preclinical [27,28,29,30] studies.

Furthermore, the current experimental results demonstrate the utility of the EcoHIV rat as a biological system to model HAND in the presence of active HIV infection. First, EcoHIV-EGFP signal was detected in the rat eight weeks after EcoHIV was infused into the brain. Data suggests, therefore, that EcoHIV infection is persistent for at least eight weeks. Second, the relative health of EcoHIV rats was evidenced by significant growth, independent of biological sex, throughout the experiment. Third, prominent neurocognitive and neuroanatomical deficits were observed in EcoHIV-infected rats within eight weeks of viral infusion. Specifically, EcoHIV-infected rats exhibited profound deficits in temporal processing, which has been suggested as an elemental dimension of HAND [32,60], as well as long-term episodic memory. Additionally, EcoHIV-infected rats displayed profound synaptic dysfunction, which may result from alterations in the NogoA-NgR3/PirB-RhoA signaling pathway, and neuroinflammation; functional alterations that may result from microglial activation. Collectively, these results emphasize the utility of the EcoHIV rat as a biological system that can be utilized to model active HIV infection as well as HAND.

The prominent neuroanatomical alterations observed in the EcoHIV rat merit further discussion given the critical need to elucidate the pathogenesis of HAND [79]. Evaluation of dendritic spine and neuronal morphology in both pyramidal neurons from layers II–III of the mPFC and medium spiny neurons from the NAc (Supplementary Figure S3) revealed prominent synaptic dysfunction. Specifically, synaptic dysfunction was characterized by a shift toward a ‘stubby’ dendritic spine phenotype (i.e., increased dendritic spine head and neck diameter) and an increased frequency of higher-order branches in EcoHIV-infected rats relative to the controls. Activation of the NogoA-NgR3/PirB-RhoA signaling pathway, which is involved in axon regeneration and synaptic destabilization [80], was evidenced by upregulation in multiple components of the signaling pathway. Critically, the binding of NogoA to NgR3 or PirB leads to the activation of RhoA and phosphorylation of PirB, ultimately resulting in actin disassembly and synapse destabilization [81], affording a potential mechanism underlying synaptic dysfunction in EcoHIV.

Neuroinflammation has been purported as a potential mechanism underlying HAND [82]. Specifically, HIV-1 infection induces NLRP3 inflammasome activation and stimulated secretion of pro-inflammatory cytokines including Interleukin-1β [83,84]. Furthermore, it has been well documented that the HIV-1 protein, Tat, can activate the NF-κB signaling pathway, subsequently priming NLRP3 inflammasomes and regulating the secretion of cytokines [85,86,87]. EcoHIV-infection induced a prominent change in various neuroinflammatory markers (i.e., IL-1β, IL-6, TNF-α, and NF-κB) in the brain relative to the control animals.

Two potential pathways by which the activation of microglia during systemic HIV-1 infection may induce neurocognitive impairments were explored (Figure 10b). First, the NogoA-NgR3/PirB-RhoA signaling pathway was activated in EcoHIV infected animals. The NogoA-NgR3/PirB-RhoA signaling pathway underlies actin assembly and thus aberrant activation of this pathway may lead to synaptic dysfunction. Second, prominent changes in various neuroinflammatory markers in the brain support the presence of neuroinflammation, which may also lead to alterations in dendritic spines, inducing synaptic dysfunction. In addition, in EcoHIV-infected rats with activated microglia, neurocognitive impairments, characterized by prominent temporal processing deficits, were observed; impairments that potentially result from dendritic spine injuries of the pyramidal neurons in layers II–III of the rat PFC. Notably, since neurons are not infected during HIV infection, damage to dendritic spines more likely results from other infected cell types (i.e., microglia). However, further studies are needed to more directly address the causal nature of these pathways.

The HIV-1 Tg and EcoHIV rat afford a complementary approach to investigating HAND by offering two biological systems with significant commonalities and distinctions. Both the HIV-1 Tg and EcoHIV rats rely on genome manipulation to offer readily available and cost effective biological systems. The expression of HIV-1 viral proteins in the CNS of both the HIV-1 Tg and EcoHIV rats supports a biological system to model key components of neuroHIV. Indeed, selective neurocognitive impairments (e.g., temporal processing, long-term episodic memory), resembling those commonly observed in HIV-1 seropositive individuals (e.g., [61,88]), have been observed in HIV-1 Tg [32,48] and EcoHIV rats; albeit neurocognitive (e.g., [31,34,89,90]) and behavioral (e.g., [74,91,92]) alterations have, to date, been more comprehensively evaluated in the HIV-1 Tg rat. Furthermore, synaptic dysfunction, a purported pathophysiological mechanism underlying HAND, is observed in both biological systems (HIV-1 Tg [33,36,93]; EcoHIV: present study). Despite these similarities, fundamental differences in the HIV-1 Tg and EcoHIV rats offer differing opportunities to expand our research in HIV-1, HAND, and associated comorbidities (e.g., drug abuse). The HIV-1 Tg rat expresses viral proteins constitutively, but does not have productive viral replication, providing a biological system to model neurodevelopmental outcomes associated with perinatal HIV-1 [94,95] and to conduct lifespan studies of HIV-1 [33,48]. The EcoHIV rat, however, is a biological system with active viral replication, offering an innovative biological system to address key understudied issues. For example, substance abuse is a significant risk factor for HIV-1, whereby injection drug use accounted for 10% of new HIV-1 diagnoses in the United States in 2018 [96]. The EcoHIV rat provides an opportunity to study the effects of prior drug history on neuroHIV outcomes. Further investigation of the EcoHIV rat will aid in the critical evaluation of its utility.

## 5. Conclusions

Collectively, the results of the present experiments identified the most prominent brain regions and cell type targeted by HIV expression in the HIV-1 Tg rat and by active HIV infection in a novel extension of the EcoHIV biological system, the EcoHIV-infected rat. In both the HIV-1 Tg and EcoHIV rats, microglia are the key cell type harboring HIV, supporting a potential target for the development of novel therapeutics. Furthermore, the EcoHIV rat, an innovative extension of the EcoHIV mouse, replicated the neurocognitive and neuroanatomical impairments described throughout the literature on HIV in clinical and preclinical studies. In conclusion, the current experiments have significant implications for our understanding of viral reservoirs and may afford a biological system to advance research on HIV-1, HAND, and the role of comorbidities (i.e., drug abuse).

## Data Availability

Data are within the article or supplementary material.

## Supplementary Materials

The following are available online at https://www.mdpi.com/article/10.3390/v13050924/s1, Table S1: Primers of neuroinflammatory factors, Table S2: Probes for the RNAscope in situ assay, Figure S1: EcoHIV distribution in cortex at seven days after stereotaxic injection, Figure S2: EcoHIV infection at seven days after retro-orbital injection, Figure S3: Dendritic spines analysis of medium spiny neurons in NAc.
