# Supplementary material for: Microglial HIV-1 Expression: Role in HIV-1 Associated Neurocognitive Disorders
Source: Viruses. 2021 May 17;13(5):924. doi: 10.3390/v13050924 (PMC8155894; doi:10.3390/v13050924)

Microglial HIV-1 expression: role in HIV-1 associated neurocognitive disorders

Hailong Li[a], Kristen A. McLaurin[a], Jessica M. Illenberger[a], Charles F. Mactutus[a], Rosemarie M. Booze [a] *

[a] *Department of Psychology, University of South Carolina, Columbia, South Carolina, USA*

*** Corresponding author:**
Rosemarie M. Booze, Ph.D.
Professor and Bicentennial Endowed Chair of Behavioral Neuroscience
Department of Psychology
1512 Pendleton Street, Barnwell College Building
University of South Carolina
Columbia, SC 29208 USA
Email: booze@mailbox.sc.edu

SUPPLEMENTARY TABLE

Table S1. Primers of Neuroinflammatory factors

Rat genes	Forward (5'-3')	Reverse (5'-3')	GenBank Identifiers
TNF-α	ACCACGCTCTTCTGTCTACTG	CTTGGTGGTTTGCTACGAC	NM 013693.3
IL-1β	GCAATGGTCGGGACATAGTT	AGACCTGACTTGGCAGAGGA	NM 031512.2
IL-6	GCCCTTGCTGGTGGATGTT	GAGAGGGAGTGCTGCTTGGA	NM 010559.3
NF-κB	GCTTACGGTGGGATTGCATT	GTTTATGGTGCCATGGGTGAT	NM_001276711.1
β-Actin	AAGTCCCTCACCCTCCCAAAAG	AAGCAATGCTGTCACCTTCCC	NM 007393.5

Table S2. Probes for RNAscope *in situ* assay

Probe	Cat. No.	Color	Comments
RNAscope® Probe-V-HIV1-CladeB-vif-vpr-tat-rev-vpu-env-nef-tar	444061-C1, ACDbio	Green	HIV-RNA
RNAscope® Probe-Rn-Aif1-C2	457731-C2, ACDbio	Red	Iba1-microglia
RNAscope® Probe-Rn-Gfap-C2	407881-C2, ACDbio	Red	GFAP-astrocyte
RNAscope® Probe-V-HIV1-CladeB-vif-vpr-tat-rev-vpu-env-nef-tar-sense	498271-C1, ACDbio	Green	HIV-DNA
RNAscope® Probe-Mm-Pirb	496031-C1, ACDbio	Red	paired Ig-like receptor B
RNAscope® Probe-Mm-Rtn4	404661-C1, ACDbio	Red	Reticulon 4 (Rtn4), Nogo A

SUPPLEMENTARY FIGURE

Figure S1 EcoHIV distribution in cortex at 7 days after stereotaxic injection

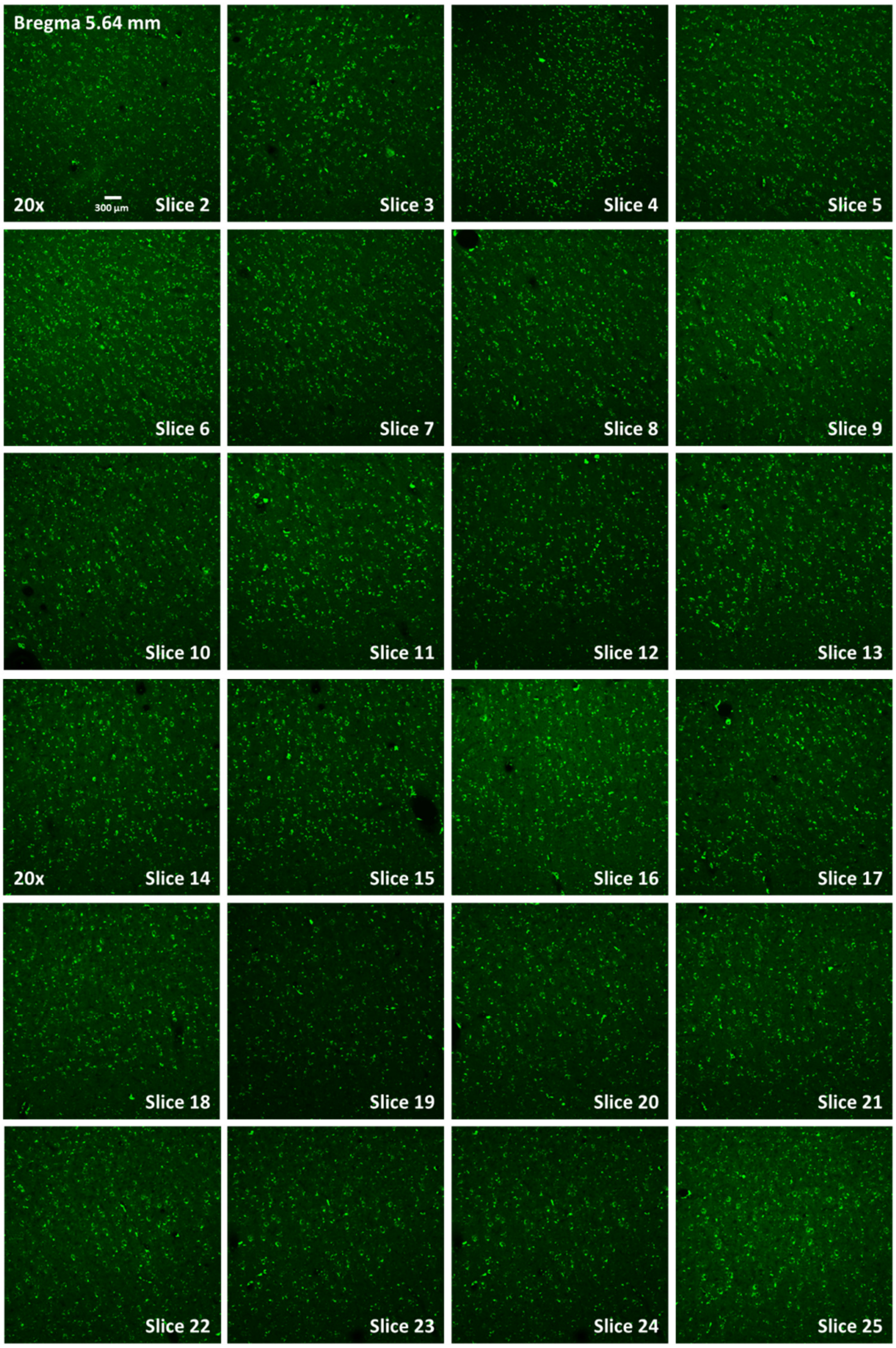

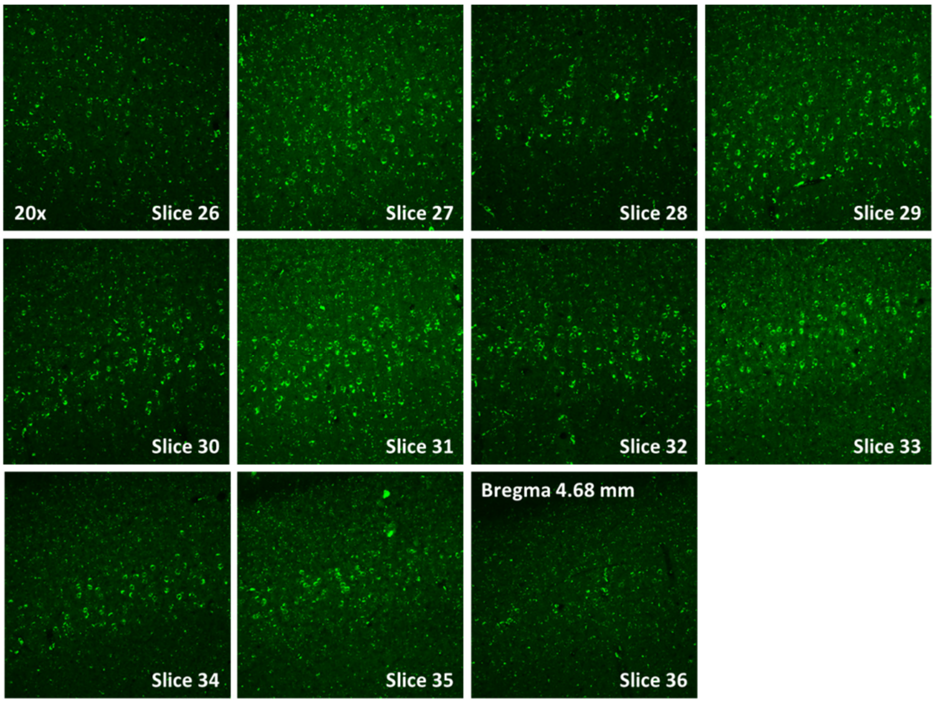

Figure S2 EcoHIV infection at 7 days after retro-orbital injection

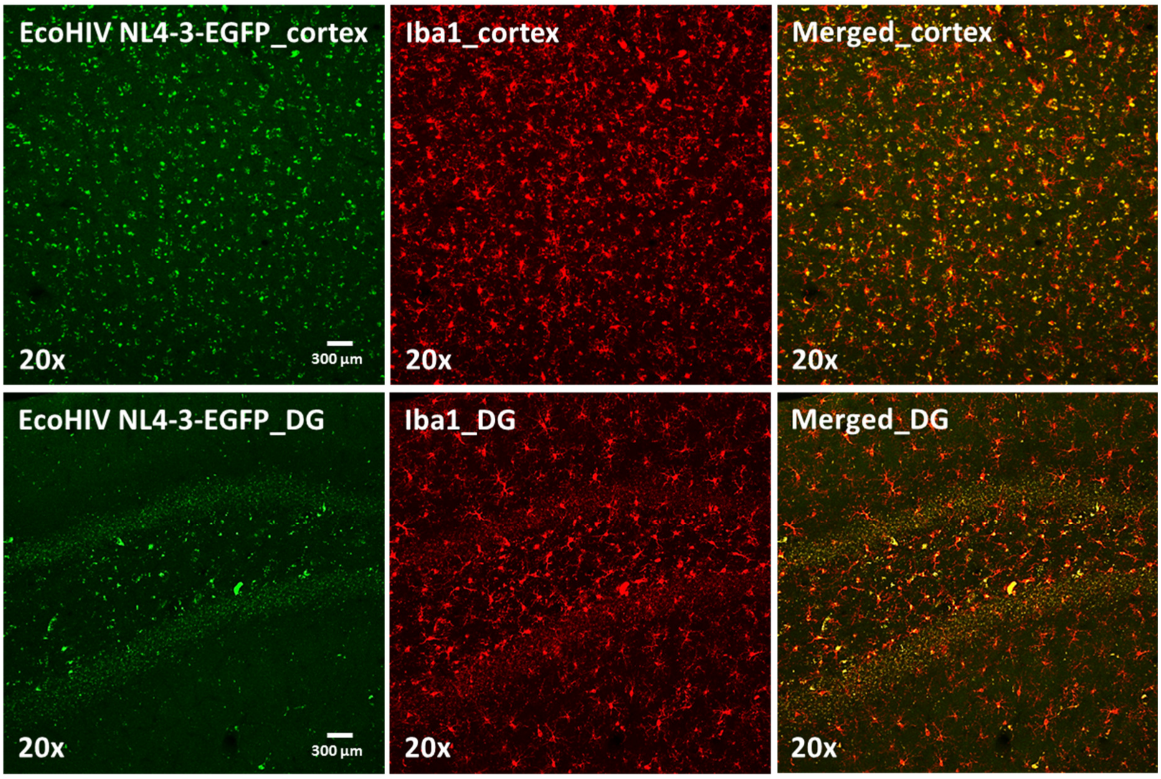

Figures S3 Dendritic spines analysis of medium spiny neurons in NAc

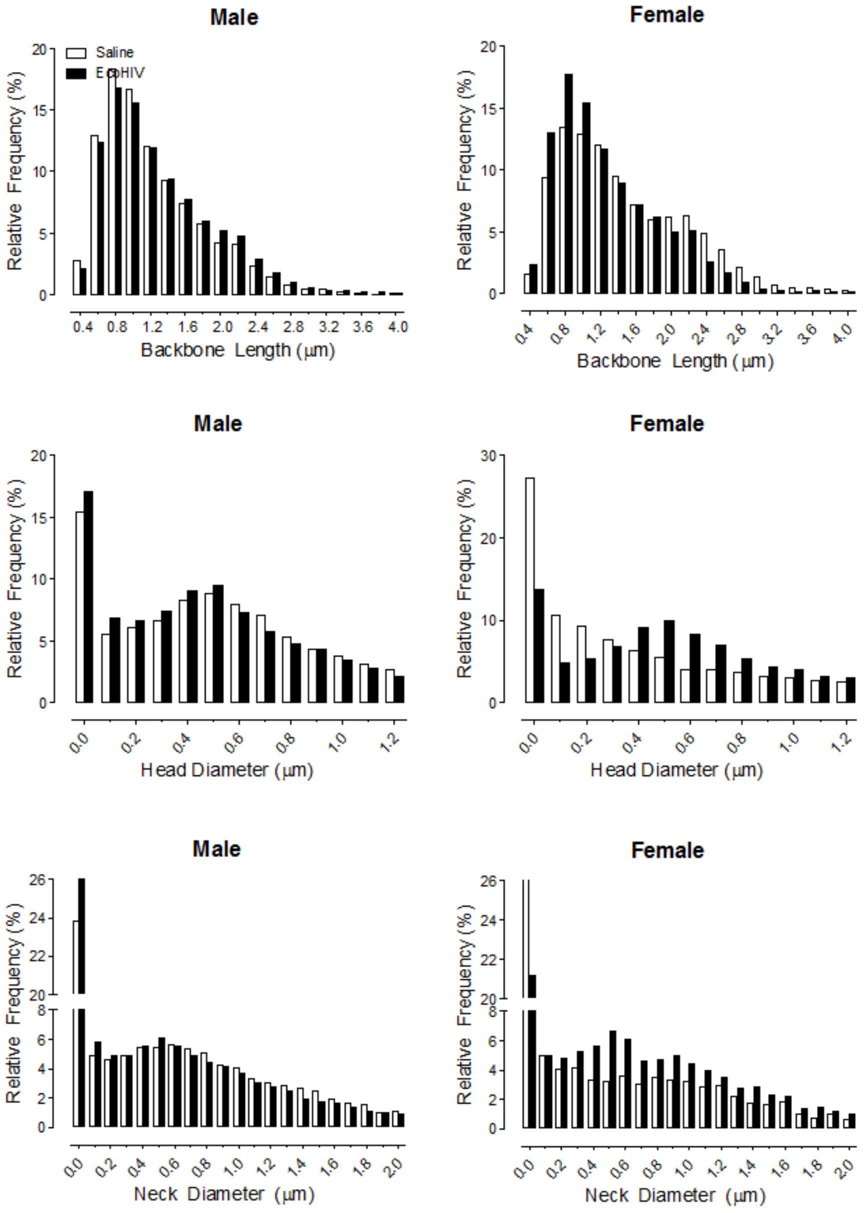

Supplement: Supplementary file 1 [file viruses-13-00924-s001.zip › viruses-1179507-SI.pdf]
